# Insecticide susceptibility status and knockdown resistance (*kdr*) mutation in *Aedes albopictus* in China

**DOI:** 10.1186/s13071-021-05095-5

**Published:** 2021-12-18

**Authors:** Yong Wei, Xueli Zheng, Song He, Xuli Xin, Jiachun Zhang, Ke Hu, Guofa Zhou, Daibin Zhong

**Affiliations:** 1 Clinical Laboratory, Shenzhen Qianhai Shekou Free Trade Zone Hospital, Shenzhen, China; 2 Department of Pathogen Biology, School of Public Health, Southern Medical University, Guangzhou, China; 3 Program in Public Health, College of Health Sciences, University of California, Irvine, CA USA

**Keywords:** *Aedes albopictus*, Deltamethrin, Resistance, kdr

## Abstract

**Background:**

*Aedes* (*Stegomyia*) *albopictus* (Skuse, 1894) is the main vector of dengue virus in China. The resistance to insecticides is a huge obstacle for the control of this species, and determining its resistance status and mechanisms in China is essential for the implementation of vector management strategies.

**Methods:**

We have investigated the larval and adult resistance status of *Ae. albopictus* to deltamethrin in eight field populations in China. Mutations at the voltage-gated sodium channel gene, related to the knockdown resistance (*kdr*) effect, were detected by sequencing of PCR products. The eight field populations were examined for pyrethroid resistance using the World Health Organization standard bioassays, and the association between the mutations and phenotypic resistance was tested.

**Results:**

The eight field populations of larvae of *Ae. albopictus* in China exhibited high resistance to deltamethrin; the RR_50_ values ranged from 12 (ZJ) to 44 (GZ). Adult bioassay revealed that *Ae. albopictus* populations were resistant to deltamethrin (mortality rate < 90%), except ZJ population (probably resistant, mortality rate = 93.5%). Long knockdown time in the field populations was consistent with low mortality rates in adult bioassay. F1534S mutation showed increased protection against deltamethrin in all populations except BJ and SJZ populations, whereas I1532T mutation showed increased protection against deltamethrin in only BJ population.

**Conclusion:**

There were different degrees of resistance to deltamethrin in field *Ae. albopictus* populations in China. The longest knockdown time and lowest mortality rate observed in *Ae. albopictus* population in Guangzhou indicate the severity of high resistance to deltamethrin. The patchy distribution of deltamethrin resistance and *kdr* mutations in *Ae. albopictus* mosquitoes suggests the necessity for resistance management and developing counter measures to mitigate the spread of resistance.

**Graphical Abstract:**

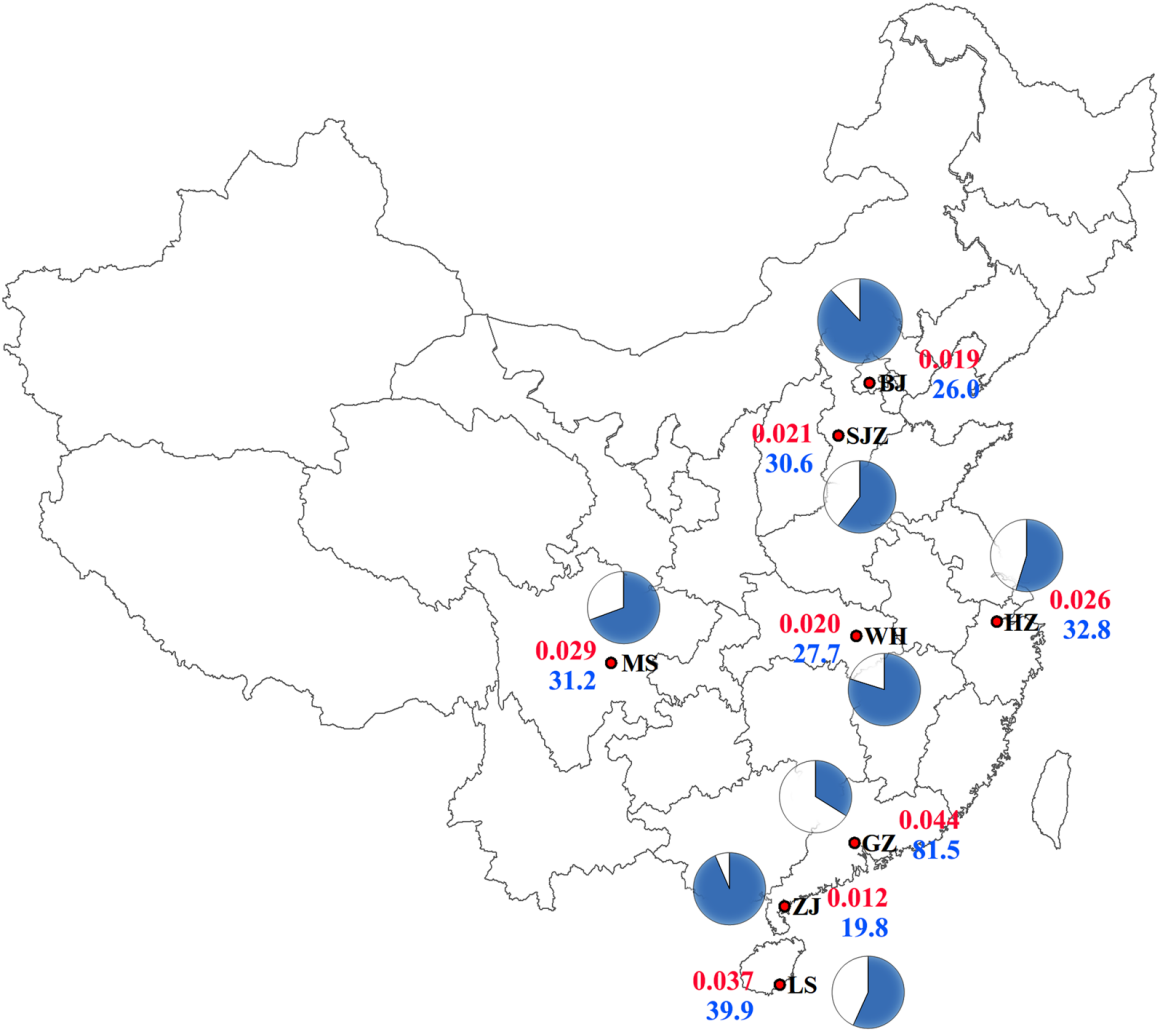

## Background

*Aedes* (*Stegomyia*) *albopictus* (Skuse, 1894) is the main vector of dengue virus in China because of its wide range in nearly one third of China from north of 41°N latitude to the southern reaches compared to the distribution of *Ae. aegypti*, limited to Hainan, Yunnan and a small area of the southernmost part of Guangdong Province [1, 2]. There were 69,321 cases of dengue reported and annual case numbers with striking variations in mainland China during 1990–2014 [3]. There were 5458 imported dengue cases distributed in 734 counties, 29 provinces and 59,183 indigenous dengue cases distributed in 314 counties and 13 provinces in mainland China during 2014–2018 [4]. The provinces affected by imported and indigenous dengue have expanded geographically from the southern to the northern parts of China [5], such as Henan Province in temperate central China where indigenous dengue cases occurred in 2013 [6]. *Aedes albopictus* is also the vector of three other important human viral diseases: yellow fever, chikungunya and Zika [7]. These diseases are a public health concern in China.

At present, due to the lack of effective drugs and vaccine for most *Ae. albopictus* transmitted parasites, except the vaccine for yellow fever virus, control of the parasites vectored by *Ae. albopictus* depends mostly on vector population control [8–10]. Reduction of larval breeding sites and use of insecticides are the major strategies for the control of vector mosquitoes [11–13]. Pyrethroids have been widely used as indoor or field sprays for mosquito control in China because of their low mammalian toxicity and rapid knockdown effect [9, 14, 15]. For example, > 27,000 kg of pyrethroids was used for ultralow-volume spraying to control adult mosquitoes during the outbreak of dengue in Guangzhou in 2014 [16]. The development of resistance is one of the main problems faced due to extensive and prolonged use of pyrethroids [15]. The populations of *Ae. albopictus* are subject to a continuous selection pressure of pyrethroids, which contributes to the fast emerging insecticide resistance [16]. It is important to monitor the susceptibility of mosquito population and understand the resistance mechanisms for controlling the local mosquito population.

The target-site insensitivity is the main resistance mechanism of *Ae. albopictus* against insecticides, in addition to increased metabolic detoxification [11, 17, 18]. The target site insensitivity results from non-synonymous mutation in the voltage-gated sodium channel (*VGSC*) gene, which has been shown to be correlated to phenotypic resistance to pyrethroids [19, 20]. The modification of VGSC protein weakens the effect of pyrethroids on the sodium channels of the nervous system via reduction or elimination of the binding affinity of the pyrethroids to proteins [21, 22]. VGSC protein is composed of four homologous domains (I-IV), of which domain III is the major one having non-synonymous mutations associated with pyrethroid resistance in *Ae. albopictus*, such as F1534S, F1534L, F1534C and I1532T [16, 19, 23–26]. In the present study, we investigated phenotypic resistance of *Ae. albopictus* adults collected from eight field populations across China and examined mutations in the partial domain III of *VGSC* gene. Moreover, the association between phenotypic resistance and *kdr* mutation was analyzed to provide complementary evidence for resistance detection of local mosquito populations at the gene level.

## Methods

### Mosquito samples

According to our previous sampling method [27], mosquito eggs were collected from breeding sites in Lingshui (LS, 110°01′59″E, 18°30′27″N), Zhanjiang (ZJ, 109°42′60″E, 21°05′37″N), Guangzhou (GZ, 113°19′42″E, 23°11′15″N), Meishan (MS, 103°52′01″E, 30°11′55″N), Wuhan (WH, 114°22′39″E, 30°30′30″N), Hangzhou (HZ, 120°07′09″E, 30°18′42″N), Shijiazhuang (SJZ, 114°27′49″E, 37°54′55″N), Beijing (BJ, 116°11′45″E, 39°51′36″N) in China from 20 July to 25 September 2019. The collected eggs were brought back to the laboratory and reared to adults (namely F0 generation) at 28 ± 1 °C and 80 ± 5% (RH), under a 16:8 h (light:dark) photoperiod. The species of *Ae. albopictus* was identified by using morphological keys as described by Lu et al. [28]. The susceptible laboratory colony of *Ae. albopictus* was collected from Foshan City in 1983 and then kept in the laboratory without insecticide exposure.

### Larval resistance bioassays

Mosquito larval resistance bioassays were conducted using deltamethrin (94.62% pure) from the Chinese Center for Disease Control and Prevention following WHO guidelines [29]. Twenty-five 3–4-instar *Ae. albopictus* larvae from F1-generation field populations were added to 99 ml of dechlorinated tap water and 1 ml of different concentrations of deltamethrin solution. Six concentration gradients, providing a range of mortalities between 10 and 90%, were tested during the experiment, three replicates per concentration. Larval mortality was recorded after 24 h exposure, and the LD_50_ values (the 50% mortality lethal concentration) were calculated.

### Adult resistance bioassays

Non-blood-fed female mosquitoes aged 3–5 days from F1 field populations and the susceptible laboratory colony were used for the resistance test against 0.03% deltamethrin following the standard WHO tube test protocol [30]. Adult bioassays were conducted with 20–28 mosquitoes per replicate and 10–20 replicates per population. The number of adult mosquitoes knocked down was recoded every 10 min during the 60-min exposure period and used to calculate the values of 50% knockdown times (KDT_50_) and 95% knockdown times (KDT_95_). Mortality was calculated after the mosquitoes were transferred to holding tubes and maintained on a 10% sucrose solution for 24 h of recovery. The dead and survived mosquitoes were collected and stored individually in 95% alcohol for subsequent DNA analysis.

### DNA isolation and PCR amplification

The genomic DNA of the mosquitoes was individually extracted using the Insect DNA Kit (Omega Bio-tek, Norcross, GA, USA), following the manufacturer’s protocol. The quality and concentration of the extracted DNA were evaluated with a NanoDrop™ 2000c spectrophotometer (Thermo Scientific, Wilmington, DE, USA). Extracted DNA was stored at −20 °C or used immediately for PCR. Partial domain III of *VGSC* gene (containing F1534 and I1532) was amplified using forward primer aegSCF7 (5′-GAG AAC TCG CCG ATG AAC TT-3′) and reverse primer aegSCR8 (5′-TAG CTT TCA GCG GCT TCT TC-3′) [19]. The PCR reaction mixture consisted of 100 ng genomic DNA, 15 μl 2 × PCR Master Mix (Promega, Madison, WI, USA), 1 μl (10 μM) forward and reverse primers, and ddH_2_O, in a final volume of 30 μl. The PCR cycling conditions were as follows: 95 °C for 5 min, followed by 35 cycles of 95 °C for 30 s, 60 °C for 30 s and 72 °C for 40 s, with a final extension at 72 °C for 10 min. The quality of PCR products was ascertained by 1.5% agarose gel electrophoresis following ethidium bromide stain. The PCR product was purified using a gel extraction kit (Omega Bio-tek) and sequenced directly with aegSCR8 using the ABI 3730XL automatic sequencer (Applied Biosystems, Guangzhou, China).

### Data analysis

The authenticity of the sequences was corroborated through the BLAST program (https://blast.ncbi.nlm.nih.gov/Blast.cgi), considering an identity percentage > 95%. The sequences were aligned and analyzed by using BioEdit v.7.2.5 software [31]. LD_50_, KDT_50_ and KDT_95_ were estimated using the log-probit models, and mortality rates were calculated in each population. A Chi-square test was used to compare differences of mortalities in adult resistance bioassays among different field populations. RR_50_/KRR_50_/KRR_95_ was calculated as LD_50_/KDT_50_/KDT_95_ of field population divided by LD_50_/KDT_50_/KDT_95_ of laboratory strain, respectively. Resistance status was classified according to WHO (2016) criteria [30]: for adult bioassays, resistant if mortality < 90%, probably resistant if mortality between 90 and 98% and susceptible if mortality > 98%; for larval bioassays, susceptible if RR_50_ < 5, moderately resistant if 5 < RR_50_ < 10 and highly resistant if RR_50_ > 10. The association between nonsynonymous mutation and the resistance phenotype was verified by Fisher’s exact test or Chi-square test, and the odds ratio (OR) was calculated for each mutation.

## Results

### Larval resistance bioassays

The eight field populations of larval *Ae. albopictus* in China exhibited high resistance to deltamethrin (Table 1). The observed LC_50_ values of field populations were 0.012 (ZJ) to 0.044 (GZ) mg/l; correspondingly, the RR_50_ values ranged from 12 (ZJ) to 44 (GZ).

**Table 1:** Resistance to deltamethrin in larval *Ae. albopictus* from eight different populations in China

Populations	LC_50_ (95% CI) (mg/l)	RR_50_^a^
Lab	0.001 (0.001, 0.001)	1.00
BJ	0.019 (0.009, 0.025)	19.0
SJZ	0.021 (0.011, 0.027)	21.0
HZ	0.026 (0.014, 0.033)	26.0
WH	0.020 (0.007, 0.027)	20.0
MS	0.029 (0.018, 0.035)	29.0
GZ	0.044 (0.036, 0.049)	44.0
ZJ	0.012 (0.005, 0.015)	12.0
LS	0.037 (0.027, 0.042)	37.0

^a^RR_50_: resistance ratio, LC50 test population/LC50 laboratory-susceptible strain

### Adult resistance bioassays

**Fig. 1:**
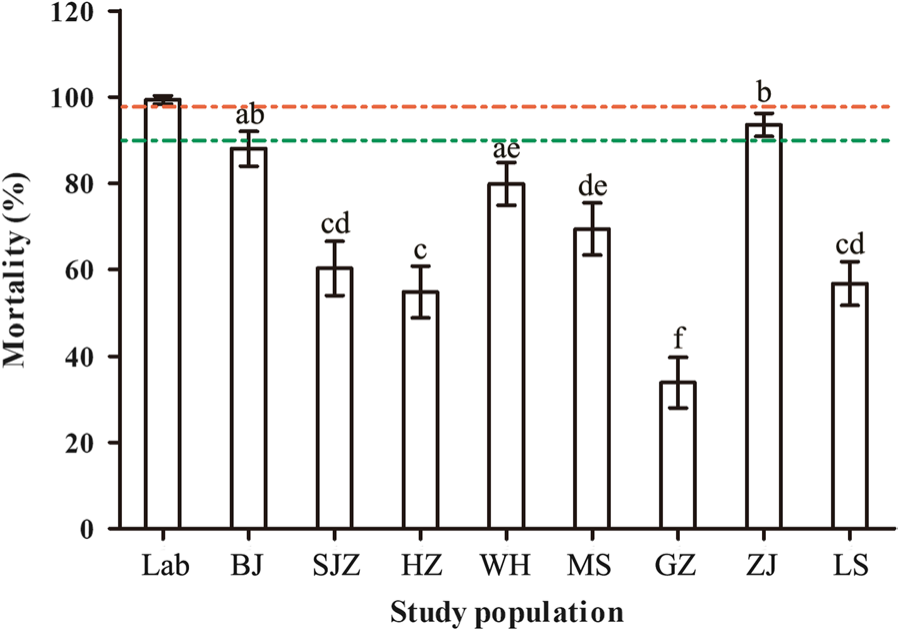
Mortalities observed with deltamethrin for the adult *Ae. albopictus* of the eight field populations and the susceptible laboratory colony. The green and red broken lines indicate respectively the mortality at 90% and 98%. Error bars indicate 95% confidential interval (CI). Different letters above bars represent significant differences between the field populations at the adjusted *P*-value < 0.00179

The eight field populations of adult *Ae. albopictus* in China exhibited resistance to deltamethrin (Fig. 1, Table 2). The highest mortality rate was observed for ZJ population (mortality rate = 93.5%, 95% CI = 90.8 – 96.2%), which was probable resistance. The other mortality rates of field populations were < 90%, with the least mortality rate of 33.9% in GZ population. A statistically significant difference was observed among mortality rates of the eight populations (χ^2^ = 338.15, df = 7, *P* < 0.001). Most pairwise differences of 28 pairs between the field populations were significant (adjusted *P*-value < 0.00179), except no significant differences between SJZ and each one of HZ, MS and LS; between LS and HZ/MS; between BJ and WH/ZJ; between WH and MS.

**Table 2:** Knockdown time and mortality rate of *Ae. albopictus* populations in China using the standard WHO tube resistance bioassay against 0.03% deltamethrin

Population	n	KDT_50_ (95% CI)	KRR_50_	KDT_95_ (95% CI)	KRR_95_	24-h mortality (95% CI)
Lab	388	18.9 (16.8–20.8)	1.0	47.6 (41.7–56.7)	1.0	99.3% (98.4–100%)
BJ	348	26.0 (23.1–28.7)	1.4	63.8 (54.8–79.0)	1.3	88.0% (84.0–92.0%)
SJZ	327	30.6 (29.3–31.9)	1.6	74.0 (68.4–81.3)	1.6	60.3% (54.0–66.5%)
HZ	362	32.8 (30.1–35.7)	1.7	84.8 (72.4–105.6)	1.8	54.8% (48.7–60.8%)
WH	343	27.7 (26.4–29.0)	1.5	76.2 (69.9–84.4)	1.6	79.8% (74.9–84.8%)
MS	305	31.2 (29.7–32.7)	1.7	87.4 (79.1–98.6)	1.8	69.4% (63.3–75.4%)
GZ	346	81.5 (72.4–96.0)	4.3	306.7 (225.5–474.9)	6.4	33.9% (28.0–39.7%)
ZJ	424	19.8 (17.3–22.2)	1.0	56.2 (48.0–69.8)	1.2	93.5% (90.8–96.2%)
LS	460	39.9 (35.1–46.2)	2.1	117.7 (88.0–198.5)	2.5	56.8% (51.7–61.9%)

KDT_50_: time in minutes when 50% of the tested mosquitoes were knocked down; 95% CI refers to 95% confidence interval

KRR_50_: knockdown resistant ratio was calculated as KDT_50_ of field population divided by KDT_50_ of laboratory strain;

KDT_95_: time in minutes when 95% of the tested mosquitoes were knocked down;

KRR_95_: KRR_95_ was calculated as the ratio of KDT_95_ of field population to KDT_95_ of laboratory strain

Lab, the susceptible laboratory colony; BJ, Beijing; SJZ, Shijiazhuang; HZ, Hangzhou; WH, Wuhan; MS, Meishan; GZ, Guangzhou; ZJ, Zhanjiang; LS, Lingshui

**Fig. 2:**
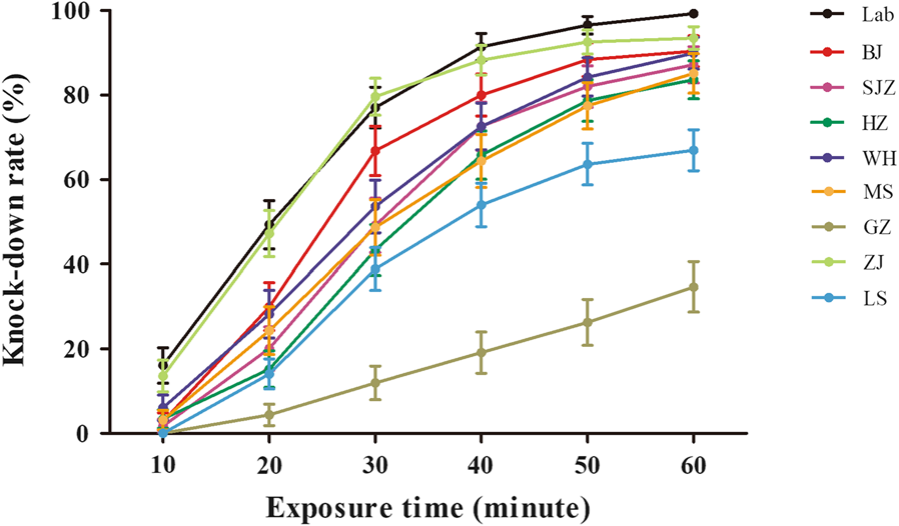
Evolution of the knockdown rates for the adult *Ae. albopictus* of the eight field populations and the susceptible laboratory colony due to exposure to deltamethrin

The knockdown time for deltamethrin showed a linear probit for knockdown rates with time in the field populations (Fig. 2, Table 2). The observed KDT_50_ values were 19.8 (ZJ) to 81.5 (GZ) min, and the observed KDT_95_ values were 56.2 (ZJ) to 306.7 (GZ) min for deltamethrin. The 50% knockdown time (KDT_50_) was 4.3 times (maximum times) in GZ population compared to the susceptible laboratory colony and nearly 1.0 times (minimum times) for ZJ population. There was a similar increase in the 95% knockdown time (KDT_95_) in field populations compared to the susceptible laboratory colony. Long knockdown time in the field populations was consistent with low mortality rates in adult bioassay.

### Detection of kdr mutations in *Ae. albopictus*

DNA was individually isolated from 18 to 24 dead and 20–24 alive deltamethrin-exposed adult *Ae. albopictus* mosquitoes in each field population. PCR amplification and sequencing of the partial domain III of the *VGSC* gene resulted in a 195-bp fragment for each study subject, with no insertions or deletions. Eight codon mutations with more than five samples were found in all the studied mosquitoes, including two non-synonymous mutations (codons 1532, 1534) and six synonymous mutations (codons 1516, 1517, 1528, 1539, 1540, 1541). At codon 1532, the mutation was detected from wild-type isoleucine to mutant-type threonine (I1532T). At codon 1534, the mutations were detected from wild-type phenylalanine to mutant-type serine (F1534S), cysteine (F1534C) or leucine (F1534L).

### Association between kdr mutations at codons and pyrethroid resistance

Among 334 *kdr* genotyping samples, 166 individuals were classified as “resistant” (being alive after the 24 h recovery period in the WHO tube bioassay) and 168 were “susceptible” (being dead after the 24 h recovery period). To determine the impact of *kdr* mutations at codons 1532 and 1534 on pyrethroid resistance, four kinds of mutations (I1532T, F1534S, F1534C and F1534L) were analyzed separately for their associations with deltamethrin resistance (Table 3). In all the populations, we found that F1534S and I1532T mutation frequency was significantly higher in the resistant compared to the susceptible mosquitoes; moreover, F1534S and I1532T conferred protection against deltamethrin, with odds ratios of 2.509 for F1534S (*P* < 0.001) and 1.864 for I1532T (*P* < 0.05). F1534S mutation showed increased protection against deltamethrin in all populations except BJ and SJZ populations, whereas I1532T mutation showed increased protection against deltamethrin in only BJ population (odds ratio = 4.200, *P* = 0.019).

**Table 3:** Association between mutations at codon 1532 and 1534 of the voltage-gated sodium channel gene and phenotypic resistance to deltamethrin in *Aedes albopictus* populations in China

Population	Phenotype	n	Odds ratio (95% CI)				*P*–value of Fisher’s exact probability test			
			F1534S	F1534C	F1534L	I1532T	F1534S	F1534C	F1534L	I1532T
BJ	R	20	na	na	na	4.200 (1.350-13.065)	0.241	na	na	0.019*
	S	20								
SJZ	R	20	na	na	3.162 (0.315-31.775)	1.202 (0.365-3.955)	na	na	0.615	1.000
	S	20								
HZ	R	20	2.786 (1.125-6.899)	na	na	1.370 (0.286-6.559)	0.043*	na	na	1.000
	S	20								
WH	R	20	3.273 (1.211-8.844)	na	na	na	0.031*	na	na	0.494
	S	20								
MS	R	20	3.316 (1.286-8.550)	na	na	0.649 (0.103-4.110)	0.021*	na	na	1.000
	S	20								
GZ	R	24	2.981 (1.194-7.446)	1.597 (0.403-6.329)	5.750 (0.538-61.409)	na	0.025*	0.722	0.279	na
	S	24								
ZJ	R	18	3.082 (1.146-8.289)	na	na	na	0.030*	na	na	na
	S	20								
LS	R	24	3.827 (1.261-11.615)	1.805 (0.591-5.513)	na	na	0.019*	0.407	na	na
	S	24								
Total	R	166	2.509 (1.789-3.518)	1.192 (0.645-2.203)	4.256 (0.848-21.358)	1.864 (1.007-3.449)	< 0.001*	0.636	0.074	0.045*
	S	168								

R, resistant; S, susceptible; na, not applicable

*P < 0.05

## Discussion

The emergence and spread of insecticide resistance are the biggest challenges to controlling vector-borne disease transmission [32]. Because many cities in China are threatened by the risk of imported dengue and local cases every year, various insecticides will inevitably be used occasionally to kill mosquitoes [33, 34]. Particularly in Guangzhou, the largest city in southern China and the epicenter of dengue outbreaks in China, the *Ae. albopictus* population has rapidly developed high resistance to deltamethrin [16, 23]. In the present study, our results showed there were different degrees of resistance to deltamethrin in field *Ae. albopictus* populations in China. Longer knockdown time and lower mortality rate were observed in the Guangzhou population of *Ae. albopictus* against deltamethrin, while shorter knockdown time and higher mortality rate in Zhanjiang population. In addition to the Zhanjiang population of *Ae. albopictus* showing probable resistance, other populations showed obvious resistance with different levels to deltamethrin. This might be related to the sampling sites in Zhanjiang from rural areas where the amount and frequency of insecticide use are relatively small compared with that in urban areas [25, 35, 36].

Rapidly emerging and widely distributed insecticide resistance is bound to affect mosquito control management and threaten the prevention and control of dengue, so it is important to develop suitable and updated guidelines for insecticide usage. It has been reported that *Ae. albopictus* larvae and adults are resistant to pyrethroid insecticides in many parts of China [37–41]. Thus, the sensitivity to current insecticides should be restored by using other highly effective and sensitive insecticides to delay the spread of resistance. For example, Su et al. suggested using malathion against adult mosquitoes and hexaflumuron or *Bti* against larvae for dengue vector control in Guangzhou [16]. Most cities’ mosquitoes in Zhejiang Province were susceptible to alpha-cypermethrin, lambda-cyhalothrin and fenitrothion, which could be used in turn to control vectors in place of highly resistant insecticides [39]. Adding piperonyl butoxide to insecticides may also be an effective formulation for resistance management [38]. High resistance to deltamethrin could lead to cross resistance to other insecticides, such as pyriproxyfen [16]. WHO recommends that rotations, mosaics and mixtures of insecticides among which there is no cross-resistance be applied in field mosquito control and insecticide resistance management [42]. Appropriate insecticides should be selected based on information obtained from insecticide resistance surveillance and used strictly following scientific guidance [42, 43].

Using the mosquito samples across China, we established that *kdr* mutation conferred protection against deltamethrin in *Ae. albopictus* by an odds ratio of 2.509 for F1534S and 1.864 for I1532T. The *kdr* mutation in the VGSC genes is one indicator of mosquito resistance to pyrethroid insecticides. I1532T and F1534S/C/L mutations have been previously reported in *Ae. albopictus* from Italy, Greece, Brazil and different areas of China [23, 24, 26, 40, 44]. The cross-continent distribution of conserved I1532T and F1534S/C/L mutations may reflect the importance of this amino acid residue in adapting to pyrethroid selection pressure. In this study, I1532T mutation was observed in BJ, SJZ, HZ, WH and MS populations, but it conferred protection against deltamethrin only in BJ population. F1534S was observed in all populations except SJZ population, and it conferred protection against deltamethrin in all populations except BJ and SJZ populations. The period in which the climate is suitable for mosquito survival is longer in southern subtropical areas than in northern areas (BJ and SJZ populations). The relatively low temperatures and dry climate in the north may not be suitable for mosquito survival, reproduction and dispersal, resulting in lower allele richness and population diversity in northern populations [45, 46]. Moreover, the frequency and duration of insecticide use in northern areas are less than those in the south [41]. In our previous study [27], it has been suggested that *Ae. albopictus* populations from southern-western China may have an evolutionary advantage over those from eastern-central-northern China. Therefore, F1534S may confer protection against deltamethrin in BJ and SJZ populations of *Ae. albopictus* in the future. F1534C was observed in GZ and LS populations, and F1534L was observed in SJZ and GZ populations, but they were not significantly associated with resistance to deltamethrin in these populations. These mutations conferring no protection against deltamethrin might be restricted by regions or affected by the number of samples tested in each region. All in all, these mutations in VGSC genes have affected or are affecting local mosquito resistance to deltamethrin in the studied populations.

We recognize several limitations in our study. First, a survey on the resistance to more insecticides would be ideal. Second, mutations of domains I, II and IV of *VGSC* gene, which were not sequenced in the present study, might affect resistance to deltamethrin, though these mutations have been reported in few studies in *Ae. albopictus* populations in China. Third, detecting the mutations of acetylcholinesterase, γ-aminobutyric and metabolic detoxification enzymes would contribute to understand comprehensively the cause of the high level of resistance to deltamethrin in *Ae. albopictus* populations in China [47, 48].

The findings of this study have important implication on the control of *Ae. albopictus*. First, it is required to monitor the insecticide resistance status and develop efficient mosquito control strategies for the patchy distribution of deltamethrin resistance in *Ae. albopictus* populations. Second, the *kdr* mutation may be a useful biomarker for pyrethroid resistance surveillance in some *Ae. albopictus* populations with a significant association between *kdr* mutation and protection from deltamethrin in China. Moreover, further work is required to solve the limitations we mentioned in this study for gaining a full understanding of the status of pyrethroid resistance and underlying mechanisms in field *Ae. albopictus* populations in China.

## Conclusions

This study reported not only the resistance status to deltamethrin in field *Ae. albopictus* populations in China, but also the association between *kdr* mutation and protection from deltamethrin. The significant association raised the possibility that *kdr* mutation may be a viable biomarker for deltamethrin resistance surveillance in *Ae. albopictus*. The patchy distribution of deltamethrin resistance and *kdr* mutations in *Ae. albopictus* mosquitoes suggests the necessity for resistance management and developing countermeasures to mitigate the spread of resistance.

## Data Availability

The data sets supporting the results are included within the article. Nucleotide sequence data reported in this paper have been deposited to the NCBI GenBank database under accession numbers OK300097–OK300430.

## Abbreviations

*kdr*: Knockdown resistance
*VGSC*: Voltage-gated sodium channel
WHO: World Health Organization
